# Dynamically changing neuronal activity supporting working memory for predictable and unpredictable durations

**DOI:** 10.1038/s41598-019-52017-8

**Published:** 2019-10-29

**Authors:** Jong Chan Park, Jung Won Bae, Jieun Kim, Min Whan Jung

**Affiliations:** 1 Department of Biological Sciences, Korea Advanced Institute of Science and Technology, Daejeon, 34141 Korea; 2 Center for Synaptic Brain Dysfunctions, Institute for Basic Science, Daejeon, 34141 Korea

**Keywords:** Cortex, Working memory

## Abstract

Diverse neural processes have been proposed as the neural basis of working memory. To investigate whether the medial prefrontal cortex (mPFC) relies on different neural processes to mediate working memory depending on the predictability of delay duration, we examined mPFC neural activity in mice performing a delayed response task with fixed (4 s) or random (between 1–7 s) delay durations. mPFC neural activity was strongly influenced by the predictability of delay duration. Nevertheless, mPFC neurons seldom showed persistent activity spanning the entire delay period and instead showed dynamically-changing delay-period activity under both the fixed-delay and random-delay conditions. mPFC neurons conveyed higher working memory information under the random-delay than fixed-delay conditions, possibly due to a higher demand for stable working memory maintenance. Our results suggest that the rodent mPFC may rely on dynamically-changing neuronal activity to maintain working memory regardless of the predictability of delay duration.

## Introduction

Working memory is a cognitive system that temporarily maintains and manipulates limited amounts of information required to perform complex cognitive tasks such as reasoning and decision making^1^. The exact neural mechanisms underlying working memory remain unclear, even though diverse candidate neural processes have been proposed^2–9^. The prefrontal cortex (PFC) is critically involved in working memory^10–12^, and early physiological studies in monkeys employing delayed response tasks have found PFC neurons that persistently maintain activity throughout the entire delay period^10,13,14^. In a typical delayed response task, a stimulus is briefly presented and then withdrawn, and a subject has to respond according to the presented stimulus after several seconds of delay. The discovery of persistent and stimulus-specific delay-period activity led to the idea that the PFC may hold information by maintaining a stable neural activity state^5,10,13^. However, diverse types of neural activity are found in addition to persistent activity in the PFC during the delay period^6,11^. In particular, in the rodent PFC, persistent activity is seldom found and, instead, sequential firing is observed during the delay period^4,15,16^, suggesting that the PFC neural network may support working memory based on dynamically-changing neuronal population activity.

Currently, it is unclear whether and how persistent and dynamic neural activity patterns contribute to working memory. Persistent activity would be useful for stably maintaining information for a prolonged time period, especially when the time of delay offset is unpredictable. By contrast, dynamically-changing activity would be useful to get ready for a proper behavioral response before delay offset. These considerations raise the possibility that a given neural system may rely more on persistent or dynamically-changing activity to support working memory according to task requirement^17^. To test this possibility, we examined whether and how the predictability of delay duration affects delay-period neural activity in the rodent medial PFC (mPFC). Specifically, we examined the possibility that the mPFC relies more on dynamic activity when delay duration is predictable, whereas it relies more on persistent activity when delay duration is unpredictable. We found that neural activity is strongly influenced by the predictability of delay duration, but delay-period activity is dominated by sequential discharges regardless of the predictability of delay duration. Our results suggest that working memory may be supported primarily by dynamically-changing neuronal population activity in the rodent prefrontal cortex.

## Results

### Behavioral performance

**Figure 1:**
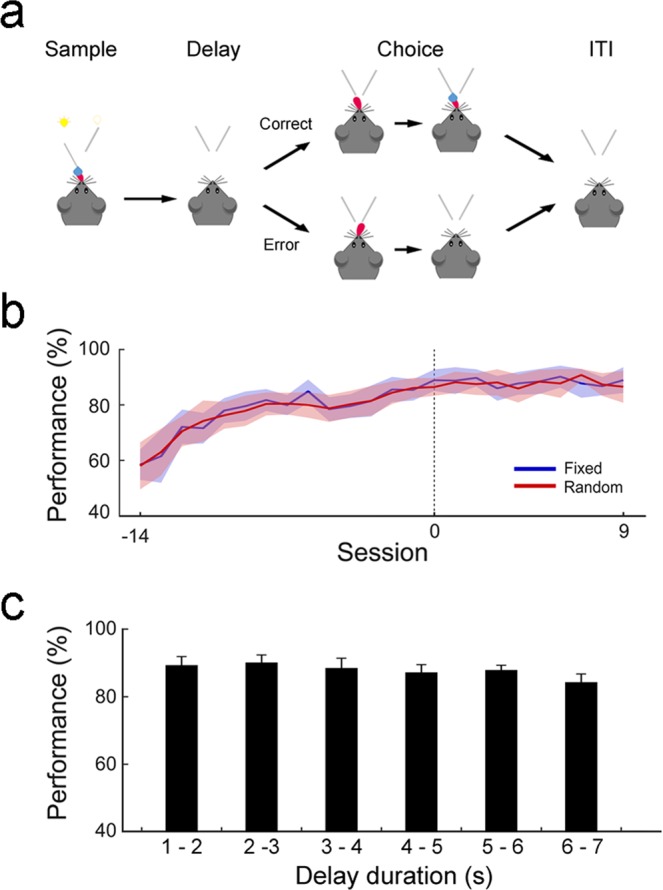
Behavioral task and performance. (**a**) A delayed match-to-sample task. A randomly chosen water port is presented and delivers water to a head-fixed mouse (sample phase). Both water ports are held retracted for a fixed (4 s) or random (1–7 s) duration (delay phase). Both water ports are presented, but water is delivered only when the animal chooses the port that delivered water during the sample phase (choice phase). Both water ports are retracted (inter-trial interval or ITI, 0–10 s). (**b**) Behavioral performance (% correct choice; mean±SEM across animals) during fixed-delay (Fixed, blue) and random-delay (Random, red) sessions. Unit recording began when the animal chose the correct target >80%. Sessions are aligned to the first unit recording session for each animal. (**c**) Behavioral performance during random-delay sessions (days 1–9) as a function of delay duration.

Twenty mice performed a delayed match-to-sample task under a head-fixed condition. The mice were rewarded with water by choosing the same target that was presented during the sample phase (Fig. 1a). A daily recording consisted of two consecutive sessions, one with the duration of delay fixed at 4 s (fixed-delay condition; 50–75 trials) and the other with the duration of delay randomized between 1 and 7 s across trials (random-delay condition; a uniform distribution between 1 and 7 s; 50–75 trials) with their orders reversed across successive days. Figure 1b shows the animal’s performance before (14 d) and during (first 9 d) unit recording. On average, the animals chose the correct target in >80% of trials under both the fixed-delay and random-delay conditions during unit recording sessions. The animal’s averaged performance did not vary significantly between the fixed-delay and random-delay conditions (two-way repeated measures ANOVA, main effect of training day, *F*(*1*, *24*) = 17.82, *p* = 7.1.2 × 10^−58^; main effect of delay condition, *F*(*20*, *960*) = 1.18, *p* = 0.28; delay condition×training day interaction, *F*(*20*, *960*) = 1.41, *p* = 0.10). In addition, the animal’s performance did not vary significantly according to delay duration under the random delay condition (one-way repeated measures ANOVA, *F*(*5*, *114*) = 0.75, *p* = 0.59; Fig. 1c).

### Examples of delay-period activity

**Figure 2:**
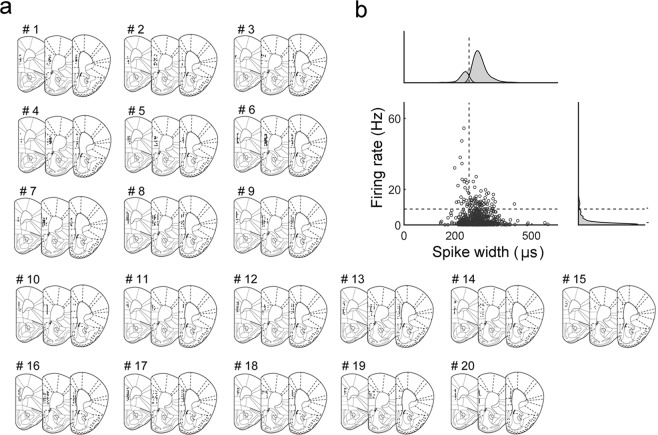
Recording locations and unit classification. (**a**) Single units were recorded from the prelimbic and infralimbic cortex. The diagrams are coronal section views of twenty mouse brains (left to right, 1.98, 1.94 and 1.78 mm anterior to bregma). Each circle represents one recording site that was determined based on histology and electrode advancement history. Modified with permission from ref.^31^. (**b**) Unit classification. Recorded units were classified based on mean discharge rates and filtered spike-waveform widths. Those units with mean firing rates <8.92 Hz and spike widths >254 μs were classified as putative pyramidal cells and included in the analysis.

A total of 1183 single units were recorded from the prelimbic and infralimbic cortex while the mice were performing the delayed match-to-sample task (Fig. 2a). The recorded units were classified into putative pyramidal neurons and putative interneurons based on mean discharges rates and spike widths (n = 861 and 322, respectively; Fig. 2b) and only putative pyramidal neurons were included in the analysis.

**Figure 3:**
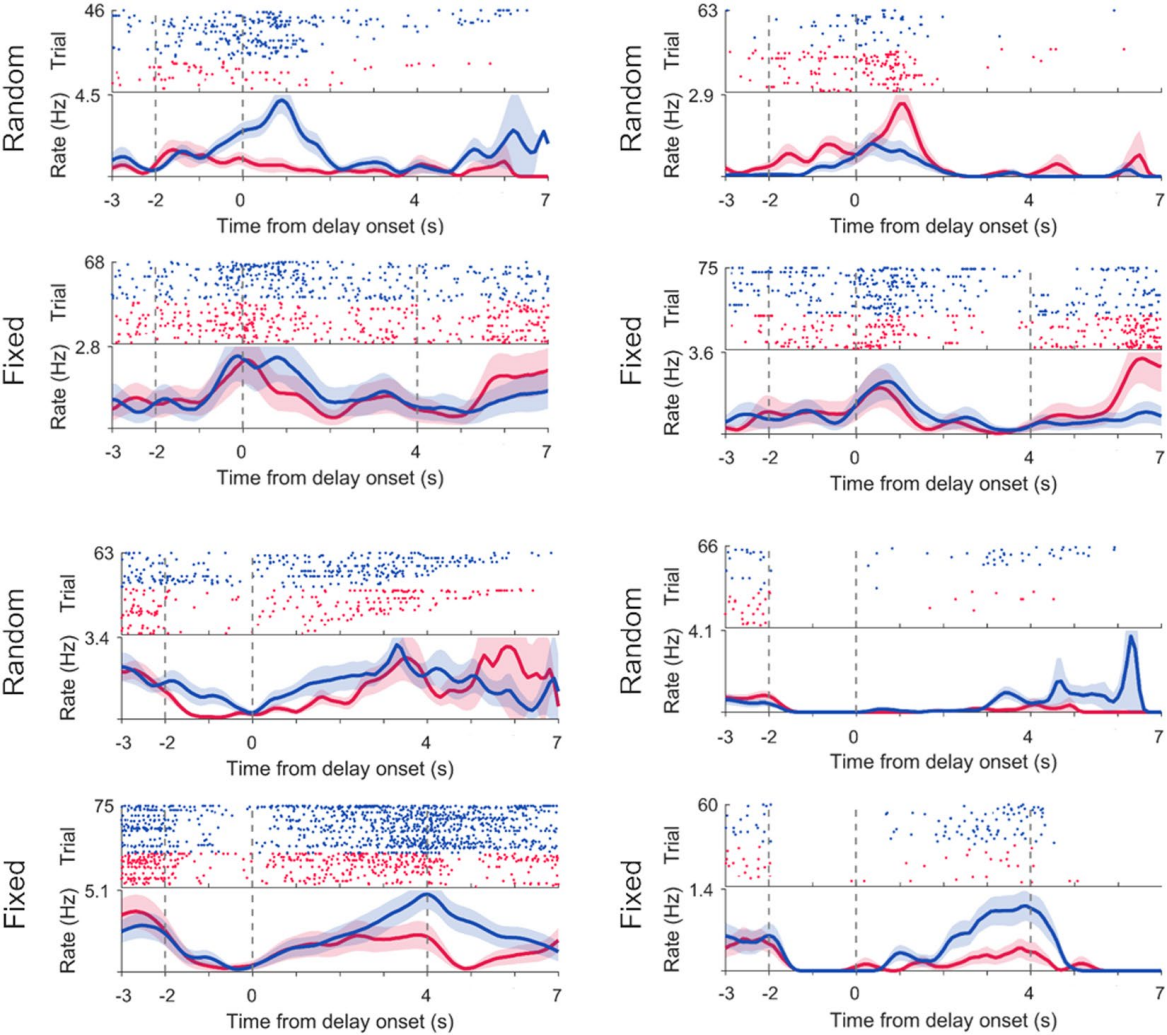
Examples of delay-period activity. Shown are spike raster plots and spike density functions (σ = 100 ms) of four example mPFC neurons. Neural activity is shown for 7 s after delay onset (fixed delay) or until delay offset (random delay). Random-delay trials are ordered according to the length of delay. Vertical dashed lines denote cue onset, delay onset and fixed delay offset (from left to right). Blue and red indicate right-choice and left-choice trials, respectively. Only correct trials are shown.

Figure 3 shows examples of neural activity during delay period. As illustrated in these examples, mPFC neurons often showed different activity patterns between left- and right-choice trials and between the fixed-delay and random-delay conditions.

### Temporal profiles of delay-period activity

**Figure 4:**
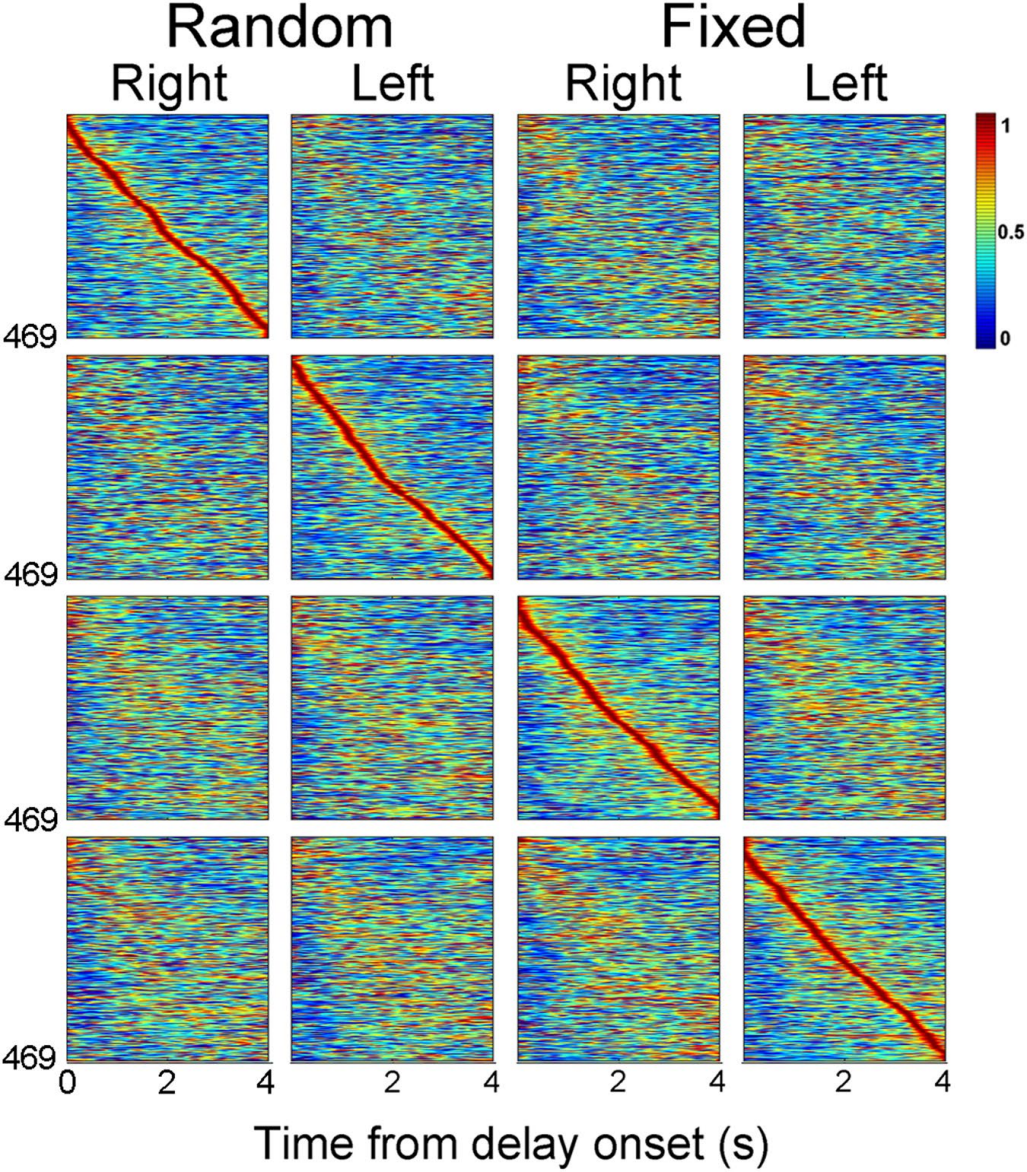
Temporal profiles of delay-period activity. Heat maps showing temporal activity profiles of all analyzed pyramidal neurons with mean delay-period activity ≥0.5 Hz during the entire fixed delay (4 s) and the initial 4 s of the random delay (n = 469). Only correct trials were analyzed and only those trials with delay durations ≥ 4 s were included and analyzed up to 4 s for the random-delay sessions. Each row represents a color-coded (red, high firing; blue, low firing; normalized to its peak firing rate) spike density function (σ = 100 ms) of one mPFC unit. Neural data obtained from the fixed and random delay conditions are shown separately. The first and second columns in each delay condition show delay-period activity during right and left target-choice trials, respectively. Neurons are ordered according to the time of peak firing under different target-choice (left vs. right) and delay (fixed vs. random) conditions. Time 0 denotes delay onset.

Figure 4 shows temporal activity profiles of all putative pyramidal neurons with mean delay-period firing rates ≥ 0.5 Hz during the entire fixed delay (4 s) as well as the initial 4 s of the random delay (n = 469; only correct trials were included in the analysis). Each row shows peak-normalized spike density functions of one unit under four different conditions that were divided according to delay type (fixed vs. random) and sample identity (left vs. right). The units were ordered according the time of peak firing under each condition. As shown, mPFC neurons tended to show different activity patterns between left- and right-choice trials and between the fixed-delay and random-delay conditions. In each condition, different mPFC units had peak firing rates at different moments during the delay period so that the time of peak firing is distributed over the entire 4-s time period.

### Task-related neural activity

In all subsequent analyses, we analyzed delay-period activity of the fixed and random delay conditions separately. For the random-delay neural data, to match the duration of the fixed-delay, we analyzed only those trials with delay durations ≥4 s and only those spikes during the initial 4 s of the delay period unless noted otherwise. Similar results were obtained when we analyzed neural activity during the last 4 s instead of the initial 4 s of the random delay (Supplementary Fig. 1). All neural data from a given delay condition (fixed or random) was analyzed together irrespective of the order of delay conditions (fixed-then-random vs. random-then-fixed) because similar results were obtained when they were analyzed separately (Supplementary Fig. 2).

**Figure 5:**
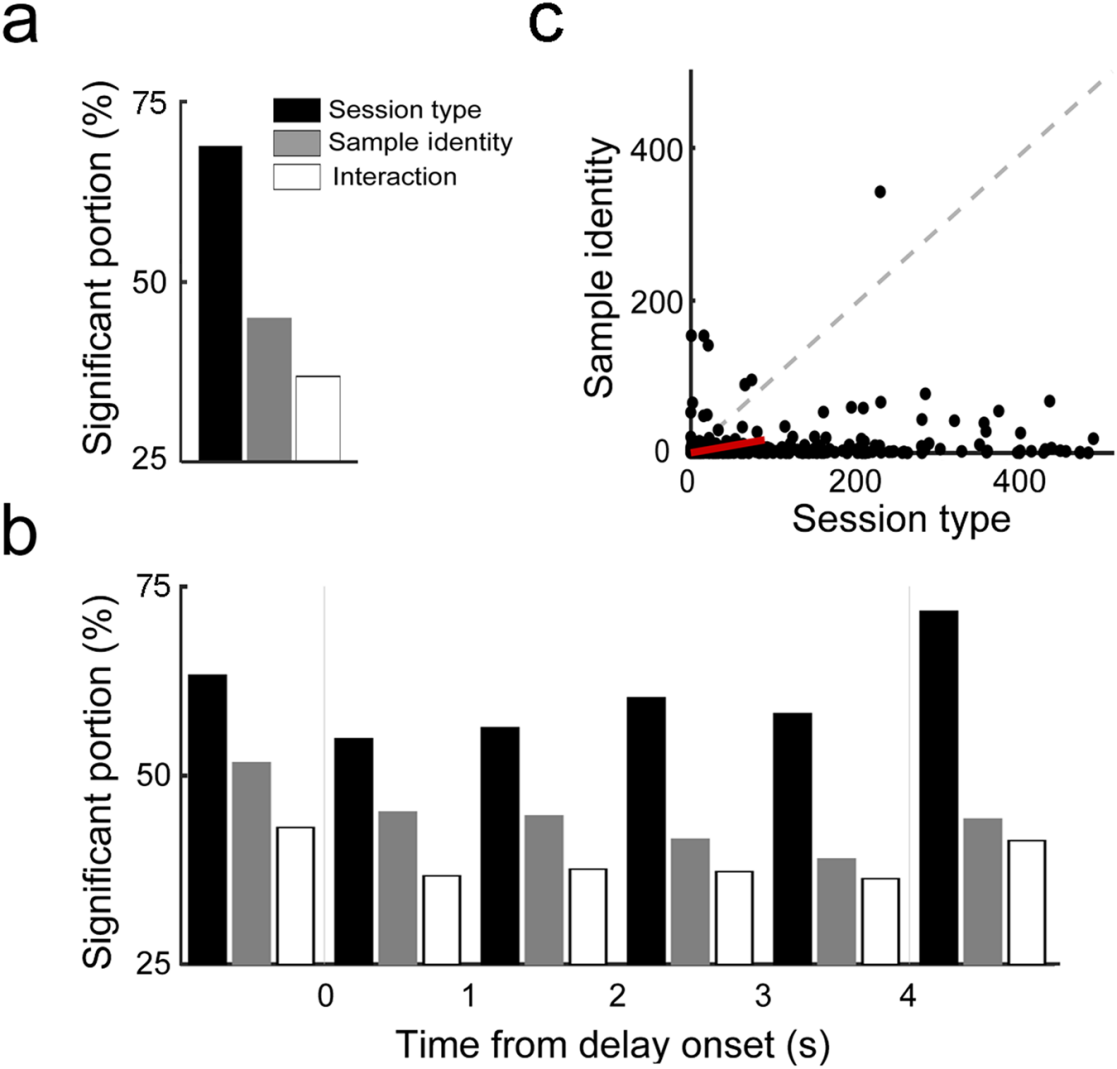
Stronger task-related than working memory-related delay-period activity. (**a,b**) Shown are fractions of neurons significantly responsive to session type (fixed vs. random delay), sample identity (left vs. right) and/or their interaction. Two-way repeated measures ANOVA was performed using neural activity during the entire 4-s delay period (the entire fixed delay and the initial 4 s of random delay; **a**) or non-overlapping 1-s time windows (**b**). (**c**) The scatter plot shows fractions of neural activity variance during the 4-s delay period explained by session type (abscissa) and sample identity (ordinate) for each neuron (*F*-values of two-way ANOVA). The red arrow is the sample mean vector.

We ran two-way repeated measures ANOVA to quantify the proportions of neurons significantly responsive (*p* < 0.05) to sample identity (left vs. right) and/or delay condition (fixed vs. random) using only correct trials. Those putative pyramidal neurons with mean delay-period firing rates ≥ 0.5 Hz during the entire fixed delay as well as the initial 4 s of the random delay were included in the analysis (n = 469). Higher proportions of units were significantly responsive to delay condition (324 out of 469; 68.9%) than sample identity (207; 44.3%; χ^2^-test, χ^2^ = 59.4136, *p* = 1.3 × 10^−14^; Fig. 5a). Similar results were obtained when we analyzed delay-period activity during non-overlapping 1-s time windows (Fig. 5b). In addition, delay condition tended to explain a larger proportion of variance in delay-period neural activity compared to sample identity (Fig. 5c). These results indicate the predictability of delay duration strongly influences delay-period activity of mPFC neurons.

### Duration of working memory information

**Figure 6:**
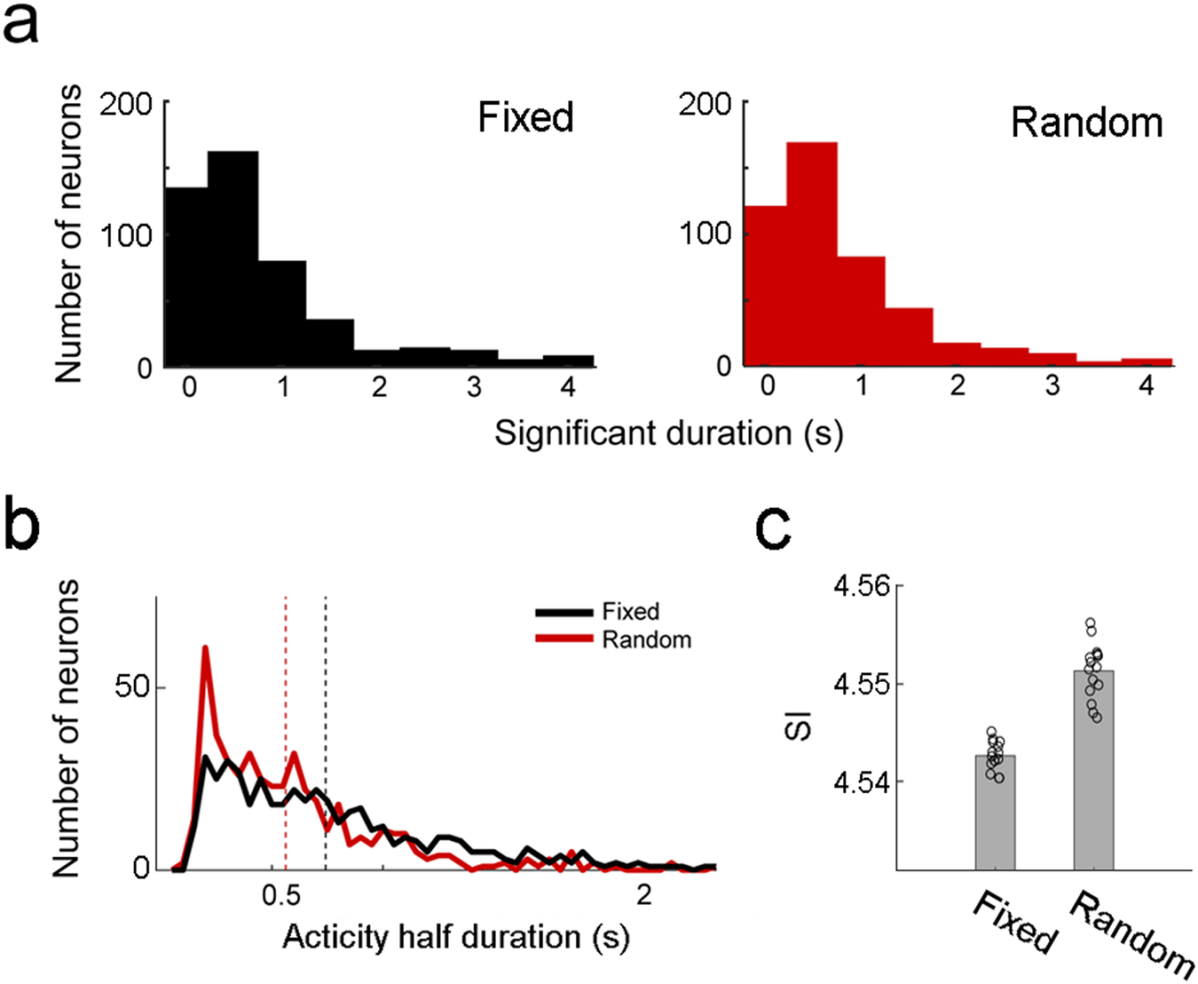
Activity duration. (**a**) The distribution of the total amount of time during which individual neuronal activity was significantly (*t*-test, *p* < 0.05) different according to the animal’s target choice (bin size, 500 ms). Only small fractions of neurons (fixed, 1.9%; random, 1.3%) were determined to maintain target-dependent activity throughout a 4-s delay period. (**b**) Distributions of activity half-duration (see Methods) for the fixed delay and the initial 4 s of the random delay. Dashed lines denote mean activity half-durations in corresponding colors. (**c**) Distributions of the sequentiality index (SI)^17^ for the fixed delay and the initial 4 s of the random delay. Circles, individual trial data (correct trials only); gray bars, their means.

To examine how many units conveyed working memory information (i.e., the identity of sample) throughout the delay period, we divided the 4-s delay period (entire fixed delay or the initial 4 s of random delay) into eight non-overlapping bins (0.5 s each) and counted how many bins have significantly different (*t*-test, *p* < 0.05) firing rates between left- and right-sample trials (correct trials only) for each unit (469 units with mean firing rates ≥ 0.5 Hz during the fixed and the initial 4 s of the random delays were analyzed). As summarized in Fig. 6a, few neurons showed significantly different neural activity between left- and right-sample trials for the entire 4-s period (fixed, n = 9 out of 469, 1.9%; random, n = 6, 1.3%). Of all neurons showing significant sample-dependent activity in at least one bin, the majority (random, 79.5%; fixed, 80.3%) showed significant sample-dependent activity in three of less bins. These results indicate that the majority of mPFC units convey working memory information only briefly during the delay period.

### Activity duration

To compare firing patterns of mPFC units between the fixed-delay and random-delay conditions, we measured activity half-durations (see Methods) of individual units using only correct trials (469 units with mean firing rates ≥ 0.5 Hz during the fixed and the initial 4 s of the random delays were analyzed). The mean (±SEM) activity duration was 742.2 ± 23.5 ms for the fixed delay and 562.3 ± 18.6 ms for the initial 4 s of the random delay (Fig. 6b). The activity half-duration was significantly longer under the fixed-delay than random-delay conditions (Wilcoxon signed-rank test, *z* = −6.948; *p* = 3.7 × 10^−12^). We obtained a similar result when we compared activity half-duration between the fixed-delay and random-delay conditions according to the time of peak activity (Supplementary Fig. 3). As another measure for sequential versus persistent firing, we examined ‘sequentiality index'^17^ which takes into account relative activity of a neuron inside a small window around its peak response time and entropy of the peak response time distribution of multiple neurons. The sequentiality index was significantly higher under the random than fixed delay condition (paired *t*-test, *p* = 1.3 × 10^−7^; Fig. 6c). These results indicate that mPFC neurons show sharper phasic firing under the random-delay than fixed-delay conditions.

### Working memory content

**Figure 7:**
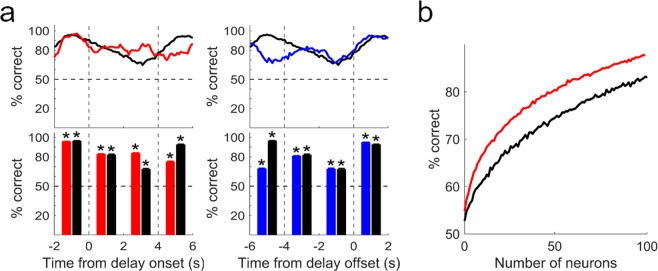
Neural decoding of sample identity. The identity of sample (left vs. right water port) was decoded based on delay-period activity. (**a**) Decoding of sample identity was based on neuronal ensemble activity (n = 90 units) during a 2-s time window advanced in 0.1 s steps (top) or during non-overlapping 2-s windows (bottom). Spikes were aligned to delay onset (red, left) or offset (blue, right) for the random delay. Black, fixed-delay. Vertical dashed lines, onset (left) and offset (right) of fixed delay. Shading and error bars, SEM across 100 times of neural decoding. *Significantly different from chance (50%) level (*t*-test, *p* < 0.05). (**b**) Neural decoding of sample identity as a function of ensemble size. Trials with delay durations ≥4 s were analyzed up to 4 s for the random-delay sessions. Decoding was based on neuronal ensemble activity during the entire 4-s period. Only correct trials were included in the analysis. Black, fixed delay; red, random delay.

To compare neural content of working memory between the fixed-delay and random-delay conditions, we decoded sample identity based on neuronal ensemble activity during the delay period in correct trials. We pooled together neurons recorded in different sessions and then decoded sample identity based on neuronal ensemble activity in each trial using a leave-one-out cross-validation procedure. We repeated neural decoding 100 times based on 90 randomly drawn neurons from each group and averaged the outcomes (% correct decoding). For the random delay, we used only those trials with delay duration ≥ 4 s and aligned spikes to the onset as well as offset of the delay. Figure 7a show the results of neural decoding in 2-s time windows (0.1-s steps) as well as non-overlapping 2-s windows (fixed delay, n = 160 units; onset-aligned random delay, n = 99; offset-aligned random delay, n = 101; see Methods for unit selection criteria). During the fixed delay, decoding accuracy decreased gradually for the initial ~3 s and then increased somewhat during the last 1 s. During the random delay, decoding accuracy was maintained more or less at a similar level throughout the delay period (Fig. 7a).

When we examined the relationship between ensemble size and the accuracy of neural decoding using the initial 4-s delay-period activity (the same set of units was analyzed; fixed delay, n = 160; onset-aligned random delay, n = 99), decoding accuracy increased as the size of ensemble increased (Fig. 7b). At an equivalent ensemble size, decoding accuracy was significantly higher under the random-delay than fixed-delay conditions (1000 times of decoding using randomly drawn neurons, ensemble size = 90 neurons, 86.8 ± 0.0 and 82.0 ± 0.0% correct decoding, respectively; *t*(1998) = 16.2795, *p* = 4.7 × 10^−56^). Decoding accuracy did not differ significantly from chance level (50%) for the pre-stimulus neural activity (1-s time period before cue onset; only correct trials with inter-trial interval >2 s were included in the analysis; ensemble size = 130 neurons, fixed delay, 48.9 ± 0.0%, *t*(99) = −0.922, *p* = 0.494; random delay, 49.4 ± 0.0%, *t*(99) = −0.610, *p* = 0.544). These results indicate that, in correct trials, delay-period activity contains a higher amount of information about sample identity under the random-delay than fixed-delay condition.

## Discussion

We investigated whether and how the predictability of delay duration affects delay-period activity in the rodent mPFC. In particular, we tested the possibility that the mPFC shows stronger persistent activity when delay duration is unpredictable. We found that the predictability of delay duration strongly influences mPFC delay-period activity in the current task. Nevertheless, mPFC neurons predominantly showed phasic, rather than persistent, activity regardless of the predictability of delay duration. Also, contrary to our prediction, working memory content was higher and phasic firing was more precisely tuned in time when delay duration was unpredictable.

Previous studies in rodents have shown that persistent neuronal activity spanning the entire delay period is seldom found in rodent mPFC, parietal cortex and hippocampus during spatial working memory tasks^4,15,16,18–20^. In these studies, the duration of delay was either fixed or the animal was allowed to navigate freely (i.e., delay duration was not imposed). In the present study, delay duration was either fixed or randomized between 1 and 7 s. Thus, under the random delay condition, the animal could not predict the time of delay offset between 1 and 7 s. Under both conditions, we found predominantly phasic discharges of mPFC neurons; we seldom found those neurons that maintain persistent activity throughout the entire delay period. These results argue against the possibility that the mPFC relies more on persistent activity when delay duration is unpredictable^17^. Even though we cannot rule out the possibility that a large fraction of neurons in the rodent brain relies on persistent delay-period activity under certain circumstances yet to be tested, such as when delay duration can be much longer than 7 s and unpredictable, our results support the possibility that working memory is maintained by dynamically changing, rather than stable, neural activity in the rodent brain regardless of the predictability delay duration. Persistent delay-period activity found in the monkey PFC may reflect a species difference in the neural basis of working memory. Primates may have evolved to equip an additional neural process (i.e., persistent activity) to support working memory, which remain to be studied.

Delay-period activity of mPFC neurons was strongly influenced by the predictability of delay duration. Compared to the neurons carrying working memory-related information, a larger fraction of neurons altered their firing rates between the fixed and random delay conditions during the delay period. This is consistent with the well-established finding that the PFC plays an important role in encoding task rules and behavioral context^21–25^. Unexpectedly, we found that mPFC neurons convey higher information about sample identity under the random-delay than fixed-delay condition. It is unclear why this is the case, but the mPFC may convey higher working-memory information because of a higher demand for stable working memory maintenance under the random delay condition. Working memory needs to be maintained only up to 4 s in the fixed delay condition, but up to 7 s under the random delay condition. Also, because it is uncertain when the delay will be terminated, it would be difficult to get ready for a behavioral response in advance under the random delay condition. Therefore, the level of working memory fidelity required to make a correct response following delay offset may be higher under the random-delay than fixed-delay condition. We also found that phasic firing was more precisely tuned in time under the unpredictable than predictable delay-duration condition. Phasic firing during delay period may be related to estimating the elapse of time^26–28^. For example, the animals may keep track of the elapse of time more precisely under the random-delay condition in an effort to predict future delay durations based on the past ones. Different levels of neuromodulatory signals associated with different levels of attention (i.e., higher attention during the random-delay condition)^29^ may underlie the observed differences in mPFC neural activity between the fixed and random delay conditions, which remains to be studied.

A caveat to our conclusions is that the unpredictability of delay duration changed over time under the random-delay condition, which may have affected neural activity. Because delay durations were uniformly (rather than exponentially) distributed between 1 and 7 s in the random delay condition, the predictability of delay duration progressively increased over time during the delay period, which may have affected our analysis results. Even though we cannot clearly rule out this possibility, we found consistently higher proportions of neurons coding delay condition than sample identity in all time windows analyzed (Fig. 5b) and narrower activity duration under the random-delay than fixed-delay conditions regardless the time of peak activity (Supplementary Fig. 3). We also failed to find a systematic change in decoding accuracy over time in the random-delay condition (Fig. 7a). These results argue against the possibility that the observed differences in neural activity between the fixed-delay and random-delay conditions are because of changes in the animal’s expectation during the delay.

## Methods

### Subjects

Twelve male *relaxin family peptide receptor 3* (*RXFP3*) mice and eight male *eighty-five requiring 3A* (*EFR3A*) mice were obtained from Mutant Mouse Resource and Research Center (CA, USA) and housed individually. They were allowed to drink water only during preforming the task and their body weights were maintained at >80% ad libitum levels throughout the experiments. All experiments were performed during the dark phase of a 12 h light/dark cycle. All experiments were performed in accordance with protocols approved by the directives of the Animal Care and Use Committee of the Korea Advanced Institute of Science and Technology (Daejeon, Korea).

### Behavioral task

The animals performed a delayed match-to-sample task under a head-fixed condition (Fig. 1a). A randomly chosen lick port was presented for 2 s during the sample phase. The animal was allowed to lick and consume water (3 μl) from the presented lick port. The presented lick port was retracted at the end of the sample phase, which marked the beginning of a delay period. Following a delay between 1–7 s, both lick ports were presented simultaneously (choice phase). The animal’s choice (licking) of the correct lick port (i.e., the lick port presented during the sample phase) triggered water (6 μl) delivery at the chosen lick port and the two lick ports were retracted after 2 s. The animal’s choice of the incorrect lick port led to the immediate retraction of both lick ports. The next trial began after an inter-trial interval (ITI; randomly selected between 0 and 10 s). The animals were initially trained in the task with the duration of delay fixed at 4 s (fixed-delay condition; 3–10 d; 150 daily trials). The animals were then trained with the duration of delay randomized between 1 and 7 s in each trial (drawn from a uniform distribution; random-delay condition) until they performed >80% correct choices for two consecutive days (2–5 days; 120–160 daily trials). They were then trained to perform the task under both fixed-delay and random-delay conditions within a day (~83 trials each) with their order reversed across successive days.

### Unit recording

A custom-made headplate was mounted on the skull and eight tetrodes and an optic fiber (diameter, 200 μm) were implanted chronically in the left or right mPFC (1.95 mm anterior and 0.45 mm lateral to bregma) under isofluorene anesthesia along with AAV2/2-EF1a-DIO-hChR2(H134R)-eYFP virus (UNC Vector Core, NC, USA) injection (0.5 μl). Unit signals were recorded from the prelimbic and infralimbic cortex (Fig. 2a). The tetrodes were lowered 50–100 μm per day once unit recording began. Unit signals were amplified (10,000×), band-pass filtered between 600 and 6000 Hz, digitized at 32 kHz, and stored on a personal computer using the Cheetah data acquisition system (Neuralynx, Bozeman, USA). Light stimuli (600 pulses, 5-ms duration, 1 Hz, 0.1–1 mW, 473 nm diode laser; Omicron Phoxx) were delivered at the end of each daily recording for optogenetic tagging of channelrhodopsin-expressing neurons. Here we report delay-period activity of all recorded units regardless of optical tagging. The results of optogenetically-tagged neurons will be reported elsewhere. Marking lesions were made by passing electrolytic current (20 μA, 15 s, cathodal) through one channel of each tetrode at the end of the last recording session. Recoding locations were verified by examining electrode tracks and marking lesions according to a standard histological procedure^30^.

### Unit classification

Putative single units were isolated by manual cluster cutting of various spike waveform parameters using the MClust software (A.D. Redish). Only those clusters with L-ratio < 0.10 and no inter-spike-interval <2 ms were included in the analysis. The isolated units were then classified into putative interneurons and putative pyramidal cells based on mean discharge rate and spike width. Those units with mean firing rates <8.92 Hz and spike widths ≥254 μs were classified as putative pyramidal cells and the rest were determined as putative interneurons (Fig. 2b). Their mean (±SD) discharge rates and spike widths were 1.5 ± 2.0 Hz and 297.1 ± 32.6 μs (putative pyramidal neurons) and 9.86 ± 8.01 Hz and 246.8 ± 36.9 μs (putative interneurons).

### Activity duration

For each unit, a spike density function was constructed by applying a Gaussian kernel with σ = 100 ms to each spike, and the resulting delay-period spike density function was normalized based on its maximum and minimum firing rates (normalized to 1 and 0, respectively). Activity half-duration was defined as the duration between the maximum (1) and half-maximum (0.5) of the normalized spike density function. When a spike density function yielded two half-durations, the longer one was used.

### Neural decoding

The identity of the sample target was decoded based on neuronal ensemble activity using the linear discriminant analysis and a leave-one-out cross-validation procedure. We included in the analysis those neurons that met the following criteria: 1) they were recorded in sessions with ≥ 15 correct-choice trials for both left and right targets; 2) their mean firing rates should be ≥ 0.5 Hz during a 4-s delay period (separately applied to the entire fixed delay, the initial 4-s of random delay, or the last 4-s of random delay); and 3) the number of 0-spike trials should be <15 for both sample targets for all analysis time windows in the 2-s sliding window analysis (Fig. 7a). We randomly selected 15 left-choice and 15 right-choice correct trials for each neuron and pooled together the resulting neural data. Then, for each correct trial (test trial), linear discriminant functions for the left and right samples were constructed based on neuronal ensemble activity during all the remaining correct trials (training trials). The identity of sample target was decoded based on neuronal ensemble activity of the test trial and the linear discriminant functions generated from the training trials assuming independence between neurons.

### Statistical analysis

Student’s *t*-tests, Wilcoxon signed rank tests, one-way repeated measures ANOVA, two-way repeated measures ANOVA, and χ^2^-tests were used for statistical comparisons. All statistical tests were two-tailed tests. A *p* value < 0.05 was used as the criterion for a significant statistical difference and all data are expressed as mean ± SEM unless noted otherwise. All analyses were conducted using MATLAB (MathWorks).

## Supplementary information

Supplementary information

## Data Availability

The datasets generated during and/or analyzed during the current study are available from the corresponding author on reasonable request.
