# Supplementary material for: Dynamically changing neuronal activity supporting working memory for predictable and unpredictable durations
Source: Sci Rep. 2019 Oct 29;9:15512. doi: 10.1038/s41598-019-52017-8 (PMC6820562; doi:10.1038/s41598-019-52017-8)
Supplement: Supplementary file 1 — Supplementary information [file 41598_2019_52017_MOESM1_ESM.pdf]

SUPPLEMENTARY INFORMATION

Park, Bae, Kim & Jung

Dynamically changing neuronal activity supporting working memory for predictable and unpredictable durations

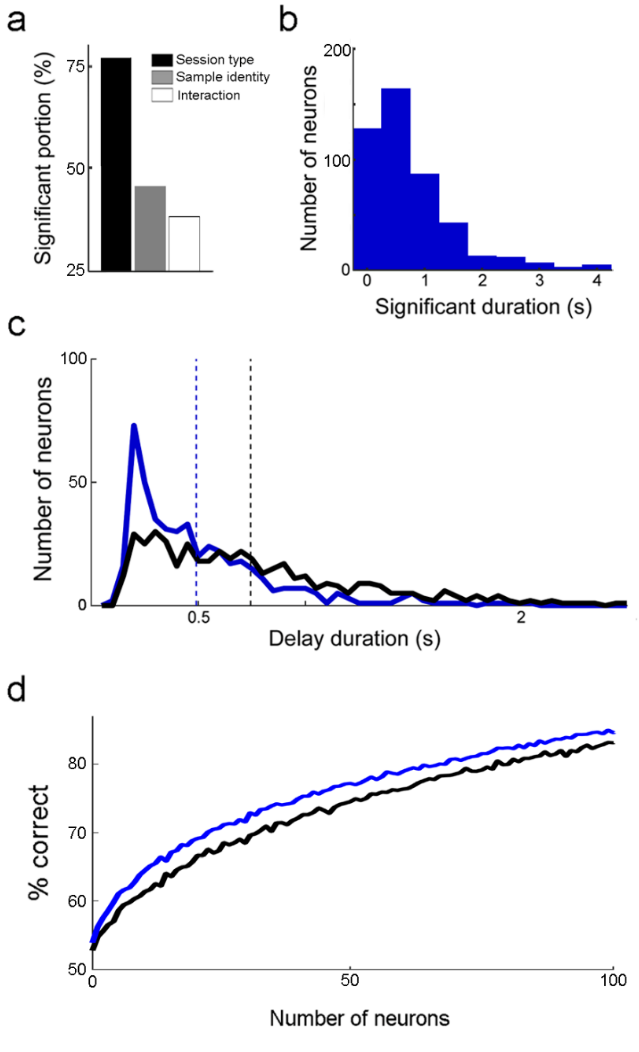

Supplementary Figure 1. Similar results were obtained with the neural data during the last 4 s of random delay. (**a-c**) Those putative pyramidal neurons with mean delay-period firing rates ≥ 0.5 Hz during the entire fixed delay (4 s) as well as the last 4 s of the random delay (n = 462; only correct trials were included in the analysis) were analyzed. (**a**) The fraction of neurons significantly responsive to session type, sample identity, or

their interaction was determined with two-way repeated measures ANOVA using neural activity during a 4-s delay period (the entire fixed delay and the last 4 s of random delay). The same format as in Figure 5a. (**b**) The distribution of the total amount of time during which individual neuronal activity was significantly different according to the animal's target choice. Neural data during the last 4 s of the random delay was analyzed. Only 5 neurons (out of 462, 1.1%) showed significantly different neural activity between left- and right-sample trials for the entire 4-s period and, of all neurons showing significant sample-dependent activity in at least one bin, the majority (n = 379, 82.0%) showed significant sample-dependent activity in three or less bins. The same format as in Figure 6a. (**c**) Distributions of activity half-duration for the fixed delay and the last 4 s of the random delay. The activity half-duration was significantly longer under the fixed-delay than random-delay conditions (Wilcoxon signed-rank test, z = -8.5507, $p = 1.2\times10^{-17}$). The same format as in Figure 6b. (**d**) Neural decoding of sample identity as a function of ensemble size. Black, fixed delay; blue, the last 4 s of random delay. We included 160 and 101 neurons in the analysis of fixed and random delays, respectively (see Methods for unit selection criteria). Decoding accuracy was significantly higher under the random-delay than fixed-delay conditions (ensemble size = 90 neurons, 83.7±0.0 and 82.0±0.0% correct decoding, respectively; *t*-test, $t(1998)$ = 5.3670, $p = 8.9\times10^{-8}$). The same format as in Fig. 7b.

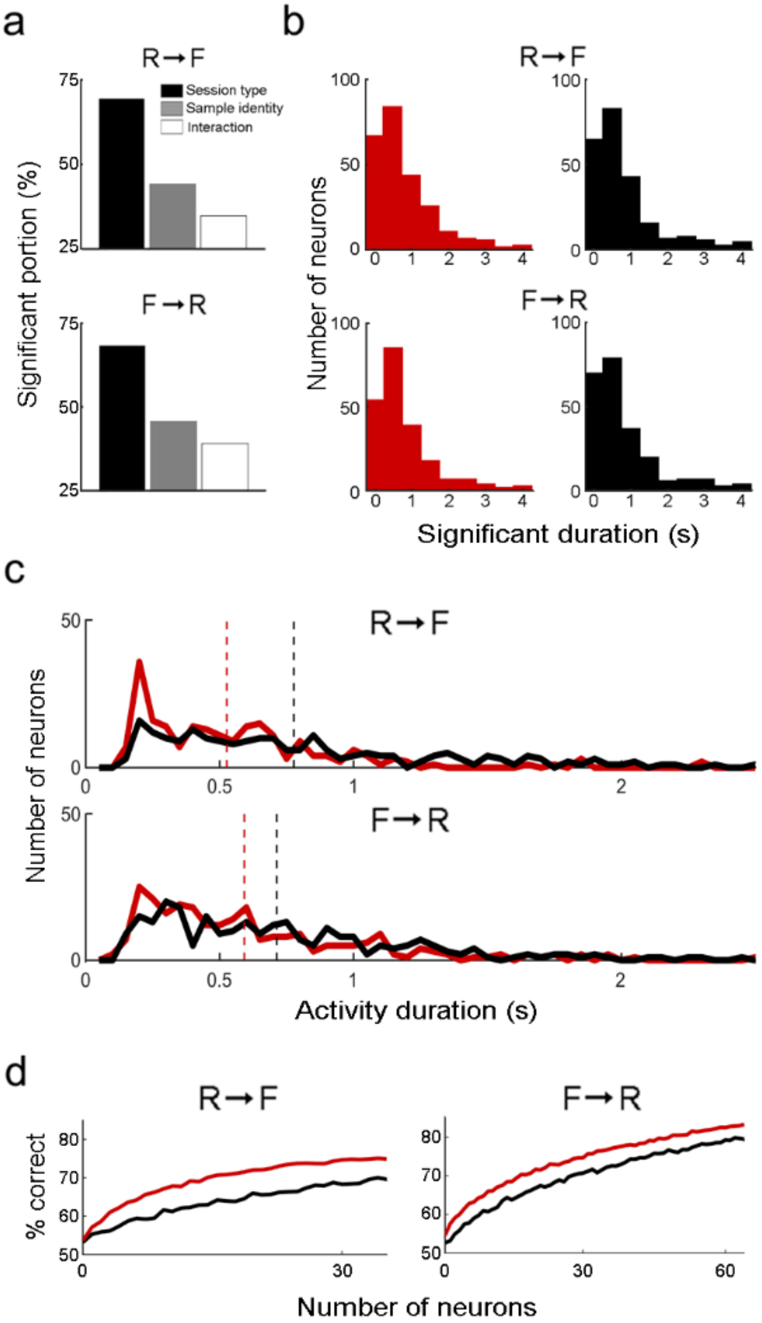

Supplementary Figure 2. Similar results were obtained irrespective of the order of delay conditions. Sessions were grouped according to the order of delay conditions: fixed-to-random (F-R) and random-to-fixed (R-F). Neural activity during the entire fixed delay and the initial 4 s of random delay was analyzed. (**a-c**) The units used in the analyses shown in Fig. 5 and 6 (n = 469) were divided into two groups according to the

order of delay conditions (R-F, n = 213; F-R, n = 256) and analyzed separately. (**a**) The fraction of neurons significantly responsive to session type, sample identity, or their interaction (two-way repeated measures ANOVA). The distributions did not differ significantly from each other (χ^2-test, $\chi^2 = 6.0$, $p = 0.1991$). The same format as in Figure 5a. (**b**) The distribution of the total amount of time during which individual neuronal activity was significantly different according to the animal's target choice. The same format as in Figure 6a. (**c**) Distributions of activity half-duration for the fixed delay and for the initial 4 s of the random delay. The activity half-duration was significantly longer under the fixed- than random-delay conditions (F-R, Wilcoxon signed-rank test, $z = -3.4675$, $p = 5.3\times10^{-4}$; R-F, $z = -6.5266$, $p = 6.7\times10^{-11}$). The same format as in Figure 6b. (**d**) Neural decoding of sample identity as a function of ensemble size. The units used in the analysis shown in Fig. 7 (fixed delay, n = 160; random delay, n = 99) were divided into two groups according to the order of delay conditions (fixed delay. R-F, n = 74; F-R, n = 86; random delay, R-F, n = 35; F-R, n = 64) and analyzed separately. Black, fixed delay; red, the initial 4 s of random delay. Decoding accuracy was significantly higher under the random-delay than fixed-delay conditions (R-F, ensemble size = 30 neurons, 74.7±0.0 and 68.3±0.0% correct decoding, respectively; *t*-test, $t(1998) = 17.8327$, $p = 4.0\times10^{-66}$; F-R, ensemble size = 30 neurons, 74.6±0.0 and 70.7±0.0% correct decoding, respectively; $t(1998) = 8.4062$, $p = 7.7\times10^{-17}$). The same format as in Fig. 7b.

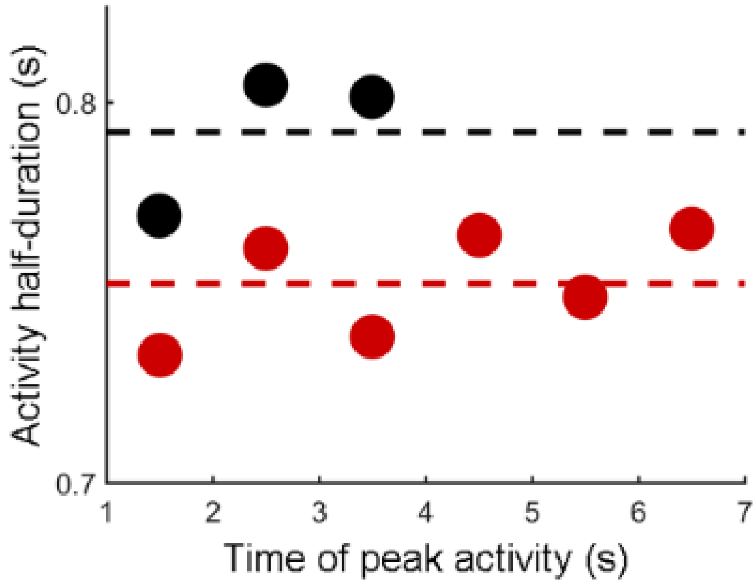

Supplementary Figure 3. Activity half-duration as a function of the time of peak activity. Circles are mean activity half-durations that are grouped according to the time of peak activity at 1-s resolution (fixed, 1-2, 2-3 and 3-4 s; random, 1-2, 2-3, 3-4, 4-5, 5-6 and 6-7 s). The same neurons used for the analysis shown in Fig. 6 were analyzed (n = 469 neurons). Dashed lines denote mean activity half-durations of all neurons.
